# Inhibiting the molecular evolution of cancer through HSP90

**DOI:** 10.18632/oncotarget.738

**Published:** 2012-11-05

**Authors:** Ana Sofia Martins, Faith E Davies, Paul Workman

**Affiliations:** Cancer Research UK Cancer Therapeutics Unit, The Institute of Cancer Research, London, UK; Divisions of Cancer Therapeutics, Molecular Pathology and Clinical Studies, The Institute of Cancer Research, London, UK; Cancer Research UK Cancer Therapeutics Unit, The Institute of Cancer Research, London, UK

**Figure 1:**
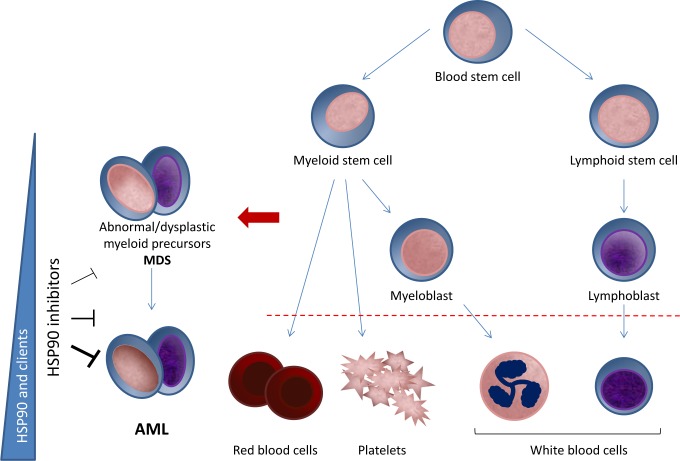
Potential for HSP90 inhibition in MDS and AML Myelodysplastic syndromes (MDS) are a group of clonal haematopoietic stem cell diseases characterised by cytopenia(s), dysplasia in one or more of the major myeloid cell lines, ineffective haematopoiesis and increased risk of developing acute myeloid leukaemia (AML). Flandrin-Gresta and colleagues (2) show that, together with its client proteins FAK, phospho-FAK and phospho-AKT, expression of the molecular chaperone HSP90 is increased in the progression of MDS into AML (left-hand side). Depleting these overexpressed and activated oncogenic client proteins (and others) with HSP90 inhibitors may be a potential novel therapeutic strategy for MDS. The thickening blunt arrows on the left-hand side indicate greater expression and potentially increasing dependence on HSP90 with progression from MDS to AML. Thus as well as having clinical potential in AML, the suggested involvement of HSP90 and its client proteins in the molecular evolution of MDS to AML suggests the possible use of HSP90 at an early stage to prevent disease progression. Such earlier use would be consistent with the hypothesis that HSP90 is necessary to support initial oncogenic transformation as well as subsequent malignant progression as a general mechanism in cancer, for example by stabilizing and preventing the proteasomal destruction of metastable oncogenic client proteins (4). Hence it is possible that the application of HSP90 inhibitors earlier in the natural history of human malignancies may have much broader clinical applicability and may reveal greater therapeutic impact.

Myelodysplastic syndromes (MDS) are a very heterogeneous group of conditions affecting the hematopoietic system. They are characterised by inefficient maturation of haematopoietic precursors with increased risk of evolution to acute myeloid leukaemia (AML) (Figure 1) (1). A new study from Flandrin-Gresta et al (2) shows the importance of the molecular chaperone heat shock protein 90 (HSP90) and its ‘client’ proteins in the molecular evolution of MDS and indicates the therapeutic potential of HSP90 inhibitors in preventing this. There are exciting broader implications for use of HSP90 inhibitors earlier in the natural history of clinical malignancies.

Standard of care for patients with MDS typically includes supportive therapy (including transfusions of the deficient cells), epigenetic drugs and/or cytotoxic chemotherapy depending on disease subtype, genetic abnormalities, International Prognostic Scoring System stage and performance status (1). Stem cell transplantation from the bone marrow of suitable donors may be a viable alternative. Treatment options were increased recently with approval of drugs such as lenalidomide, and there are other new agents and drug combinations currently undergoing clinical evaluation (1). Nevertheless, novel agents targeted to the molecular pathogenesis of MDS are urgently needed.

Many molecular aberrations occur in MDS, ranging from chromosomal abnormalities, amplifications, deletions and mutations to epigenetic changes. Genes involved cover a variety of biological processes supporting malignancy and include *TET2, ASXL1, RUNX1, IDH1, IDH2, EZH2*, and those encoding various tyrosine kinases, as well as *NRAS, KRAS, CBL and TP53* (3).

The importance of HSP90 in cancer is well established, in particular its role in supporting the active conformation of many oncogenic client proteins (4). Pharmacologic inhibition of HSP90 causes degradation of these client proteins by the ubiquitin-proteasome pathway, profoundly inhibiting many signal transduction pathways and inducing cell cycle arrest and apoptosis (5). The therapeutically attractive ability of HSP90 inhibitors to deplete simultaneously many cancer-causing clients, reducing the molecular avenues via which drug resistance can develop, has led to these agents becoming of major pharmaceutical company interest, with ~20 inhibitors entering clinical trials (4).

Phase I and II clinical studies have shown promising results for HSP90 inhibitors in malignancies addicted to particular HSP90 clients, especially HER2+ trastuzumab-refractory breast and EGFR-mutant and ALK-translocated non small cell lung cancer, as well as other opportunities in haematological cancers, including multiple myeloma (where HSP90 inhibition exploits the unfolded protein response) and leukaemias driven by HSP90 clients (eg FLT3 in AML and BCR-ABL in chronic myeloid leukaemia) (4).

Previous studies in a large series of AML patients revealed an association between higher expression levels of HSP90 and poor prognosis together with enhanced activation of oncogenic signaling pathways involving PI3 kinase, AKT and ERK1/2 (6). Furthermore, HSP27, HSP70 and HSP90 are overexpressed in advanced MDS compared to early MDS and normal bone marrow, consistent with involvement of HSPs in the pathogenesis and evolution of this condition (7).

In the present issue of Oncotarget, Flandrin-Gresta and colleagues now report the clinical and biological relevance of HSP90 and phosphorylated forms of its protein kinase clients AKT and FAK (focal adhesion kinase) in bone marrow mononuclear cells (dysplastic white cells) and CD34+ cells (myeloid stem cells) from a series of 177 patients with MDS, determined at diagnosis and in some cases after evolution to high grade MDS or to AML (2).

First, they observe that HSP90, FAK, phospho-FAK and phospho-AKT are overexpressed in high risk MDS and are associated with shorter survival and increased risk of progression to AML. The expression levels of all of these proteins were significantly higher after transformation than at the time of diagnosis.

Next, the authors show that the pharmacologic HSP90 inhibitor tanespimycin (17-AAG) reduces viability and markedly increases apoptosis in bone marrow mononuclear cells and CD34+ cells isolated from 39 patients. Furthermore, tanespimycin decreases expression of FAK, phospho-FAK and phospho-AKT. Somewhat surprisingly, HSP70 is not induced while HSP90 levels are decreased.

Taken together, these results suggest a possible new clinical application for HSP90 inhibitors in MDS, either alone or combined with agents such as disease-relevant kinase inhibitors or epigenetic therapies (8). In addition, HSP90, phospho-FAK and phospho-AKT may be useful prognostic or predictive biomarkers in MDS.

Further work is needed to understand the exact mechanisms by which HSP90 is involved in the pathogenesis of MDS and its progression to AML, including the contribution of chaperone support for FAK, AKT and quite possibly other HSP90 clients, including mutated FLT3. In addition, following termination of tanespimycin development, preclinical and clinical studies are now needed to assess the impact of improved, next-generation HSP90 inhibitors (4) in MDS.

Of particular biological as well as therapeutic interest is that the use of HSP90 inhibitors to prevent the molecular evolution of MDS to AML would be the first example of their application to prevent progression from pre-malignant to fully malignant disease. Such a clinical use, potentially applicable to other cancers, would be consistent with the hypothesis that HSP90 is essential to support initial oncogenic transformation as well as subsequent malignant progression, for example by preventing destruction of metastable oncogenic driver client proteins. This would encourage treatment with HSP90 inhibitors earlier in the natural history of other human malignancies, with potential for even greater clinical impact.
